# Gender-specific treatment effects and outcomes reported in orthodontic research. A cross-sectional empirical study

**DOI:** 10.1093/ejo/cjad073

**Published:** 2023-12-10

**Authors:** Géraldine Kummer, Theodore Eliades, Despina Koletsi

**Affiliations:** Clinic of Orthodontics and Pediatric Dentistry, Center of Dental Medicine, University of Zurich, Zurich, Switzerland; Clinic of Orthodontics and Pediatric Dentistry, Center of Dental Medicine, University of Zurich, Zurich, Switzerland; Clinic of Orthodontics and Pediatric Dentistry, Center of Dental Medicine, University of Zurich, Zurich, Switzerland; Meta-Research Innovation Center at Stanford (METRICS), Stanford University, Stanford, CA, United States

**Keywords:** gender-specific effects, sex-sensitive medicine, orthodontic outcomes, gender-sensitive medicine, subgroup analysis

## Abstract

**Aim:**

To identify practices of assessment of gender effects in research articles in orthodontics and detect whether there were significant differences in the treatment effects on outcomes according to gender.

**Materials and Methods:**

Four major orthodontic journals were sought over a 3-year period to identify publications which included assessment of gender effects on outcomes in their reporting. Data were extracted on the following characteristics: journal, year of publication, region of authorship, and study design. For the studies including reporting of gender effects, whether a significant effect existed was further documented. Additionally, for these studies, data were extracted on population, sample size per gender, treatment, comparison, outcome type, and nature and whether gender analysis was based on subgroup testing or included as a main effect. Descriptive statistics, cross-tabulations, univariable, and multivariable regression models were utilized as appropriate.

**Results:**

A total of 718 research articles were eligible for inclusion out of a pool of 1,132 screened articles. Of those, 95 reported on any type of analysis on gender effects (95/718; 13.2%). In the 95 studies that reported assessment of gender effects, it was clear that the majority did not detect significant gender-related differences across the documented outcomes (range of frequency distribution for significant gender differences across all outcomes: 0–50%). Twenty-two articles overall (22/95; 23.2%) described a significant gender effect classified by outcome, 12 favoring female and 10 favoring male participants. Patterns of efficacy and adverse outcomes were schemed either favoring female (root resorption: 4/10; 40.0%, periodontal outcomes: 3/11; 27.3%) or male (cephalometric/growth changes following orthodontic treatment: 4/17; 23.5%) patients across the 22 studies with significant effects. Appropriately designed and adequately powered statistical analyses, with gender effect assessment as a main effect in a multivariable regression model was associated with 6.53 times higher odds for identifying significant gender effects (OR = 6.53; 95% CI: 2.15, 19.8; *P* = .001).

**Conclusions:**

A very small proportion of research studies included gender effect assessment in their analyses. Of those, a quarter described significant effects. Nevertheless, careful analysis planning and strategies should be prioritized to allow for any meaningful interpretation.

## Introduction

“Precision” or “personalized” medicine has been a longstanding concept that has gained an increasing interest in recent years. It is expected to gain a wide adoption of its standpoints in the years to come, fueled by the exploitation of the technological advancements, machine learning, and development of artificial intelligence [1]. What these terms convey is represented by a swift tailoring of therapeutic approaches built on characteristics of individuals or subgroups of individuals, given shared genetic, epigenetic, societal, lifestyle, and other susceptibility-related features. These make them correspondingly prone to develop a common pathway of disease or shared responses to therapeutic interventions [2, 3]. The precision medicine model shift in healthcare offers the perspective of informed decision making based on unique individual characteristics, rather than evidence derived from the average patient [1, 4].

Allied to the pillars of precision medicine, the concept of gender-specific treatment approaches and effects of interventions is becoming compelling, to chart differences and interpret variability in outcome ascertainment, based on gender. Specifically, the developed terminology “sex- and gender- sensitive medicine” describes both the biological as well as the societal and environmental effect of sex and gender respectively, on interpretation of diseases, treatment strategies, and outcomes [5, 6]. For simplicity reasons, the present article will use the term “gender” throughout, as a collective term for the above. As such, failure to integrate gender-specific data and evidence at all healthcare levels may result in compromised health outcome solutions, inadequate treatment modalities for either male, female, or both, and even distorted clinical decision making. Subsequent consequences may impact research translation and reproducibility of scientific evidence. This does not exclude oral healthcare, dental health issues, and orthodontics from similar considerations. It has been speculated that male patients tend to underestimate the importance and interrelation of oral health to general health and systematic diseases, are more likely to experience periodontal disease and dental trauma, or have poorer oral hygiene status, being also less proactive in visiting dentists [7, 8]. Firm evidence regarding the variability in effects of gender on the perceived outcomes of a given oral health intervention would promote the development of more efficient treatment strategies and would also assist in the advancement of early diagnosis perspectives.

Considering the above, editorial policies of major scientific journals have embraced the need for publication of research findings in a model of gender-disaggregated data [5, 9, 10]. Reporting guidelines and recommendations for gender-related reporting strategies have been proposed for data analysis, results, and interpretation of findings [11]. Funding agencies have been also moving to a similar direction. The Canadian Institutes for Health Research has adopted gender integration in health research build-in grant and funding opportunities [12–14]. As an essential first step to facilitate documentation of the state of the art that will dictate the need for further action, one could map existing evidence on original research findings according to gender; then identify outcome specific, gender-related treatment effects.

To our knowledge, there is yet no similar attempt related to orthodontic research. It was therefore the aim of the present cross-sectional empirical study first, to identify published orthodontic research that provided gender data on treatment outcomes and second, to assess whether these outcomes related to significant differences in gender-specific treatment effects, between male and female patients. Furthermore, potential predictors of identification of gender-specific treatment effects were also explored.

## Materials and methods

We electronically searched the contents of four major orthodontic journals to identify publications involving treatment effects and being on paper for a reasonable period back in time, to determine gender impact on outcomes of otherwise commonly utilized interventions. We included observational (cohort, case control) and interventional (prospective clinical trials, randomized clinical trials) studies published between 1 January 2018 to 31 December 2020 in the following major specialty journals: The *American Journal of Orthodontics and Dentofacial Orthopedics* (*AJODO*), the *Angle Orthodontist* (*ANGLE*), the *European Journal of Orthodontics* (*EJO*), and the *Progress in Orthodontics* (*PIOR*). All article types that pertained to non-research articles, such as editorials, letters to the editor, commentaries, and opinions, were *a priori* excluded and did not count toward the pool of potentially eligible articles. On a second stage, article types such as systematic reviews/meta-analyses, case reports, *in vitro*/*in silico* studies, meta-epidemiologic studies, cross-sectional studies not assessing treatment effects, case reports, technique description articles, or animal studies were further excluded.

Data extraction was performed on piloted standardized forms following initial calibration procedure between the two assessors of the study (G.K. and D.K.) in 40 articles. Any preliminary disagreement on piloting was resolved through discussion and achievement of consensus between the two investigators. We extracted data on the following variables: journal, year of publication, region of authorship in geographic terms (based on affiliation of first author), and study design type (observational or interventional). On an outcome level, we recorded whether the article assessed gender-specific effects on respective outcomes.

For the sub-sample of studies that reported gender-specific assessments, we further documented whether the treatment effects differed in a significant level between male and female patients. Additionally, for these studies, we also recorded the population (children including adolescents, adults, mixed), the sample size based on gender stratification, the treatment provided, the comparison (active comparator group(s), untreated control, or no comparison) and the outcome, both in terms of nature (efficacy, adverse) as well as in terms of actual outcome (i.e. root resorption, tooth movement/ duration, relapse, compliance, etc.). We further documented whether analysis of gender were based on subgroup analyses or comprised the main objective of the study and/ or were included as effects in a multivariable model.

### Statistical analysis

Descriptive statistics and cross-tabulations were initially conducted to present characteristics of the sample of studies examined regarding the aforementioned variables and also with regard to the sub-sample of studies identified as those including an assessment of gender effects. Univariable and multivariable logistic regression was performed to examine the effect of publication characteristics including journal, year of publication, region of authorship, and study design type, on performing gender-specific analyses. In addition, univariable and multivariable logistic regression was performed to assess whether a significant gender effect (either favoring males or females) related to study design type, analysis type (main, subgroup), population (children including adolescents, adults, mixed), and outcome (efficacy, adverse).

Potential predictors were sequentially introduced to the model (forward stepwise variable selection in order of significance). To balance model fitness and its complexity, the Akaike Information Criterion was assessed to structure and select the final multivariable regression model. The Hosmer–Lemeshow test was used to check model fit.

On an exploratory basis, for the sub-sample of studies that reported implementation of gender-specific assessments, we recorded the descriptives of female/male distribution of participants according to gender in the included studies and further tested for actual differences in distributions through the Wilcoxon signed rank test, following assessment of normality assumptions for the data (non-parametric). In addition, the frequency distribution of gender across the study samples was checked through chi-square as being even or uneven. A criterion of 10% difference was established for this reason, as follows: a 10% difference in gender enrollment (sample size) was allowed to account for an approximately equal distribution. Exceeding this threshold was indicative of uneven gender distribution.

The unweighted κ statistic was used to assess inter-rater agreement on the pre-determined predictor variables. An almost perfect agreement was deemed across the variables ranging from κ = 0.92 (95% confidence interval [CI]: 0.73, 1.00) to κ = 1.00. For the recording of variables related to the sub-sample of studies which included a gender-specific analysis, all recordings were performed independently by both investigators and consensus was achieved. The predefined level of significance was set at *P* < .05 (two-sided). All analyses were conducted with Stata version 15.1 (Stata Corporation, College Station, TX, USA).

## Results

**Figure 1.**
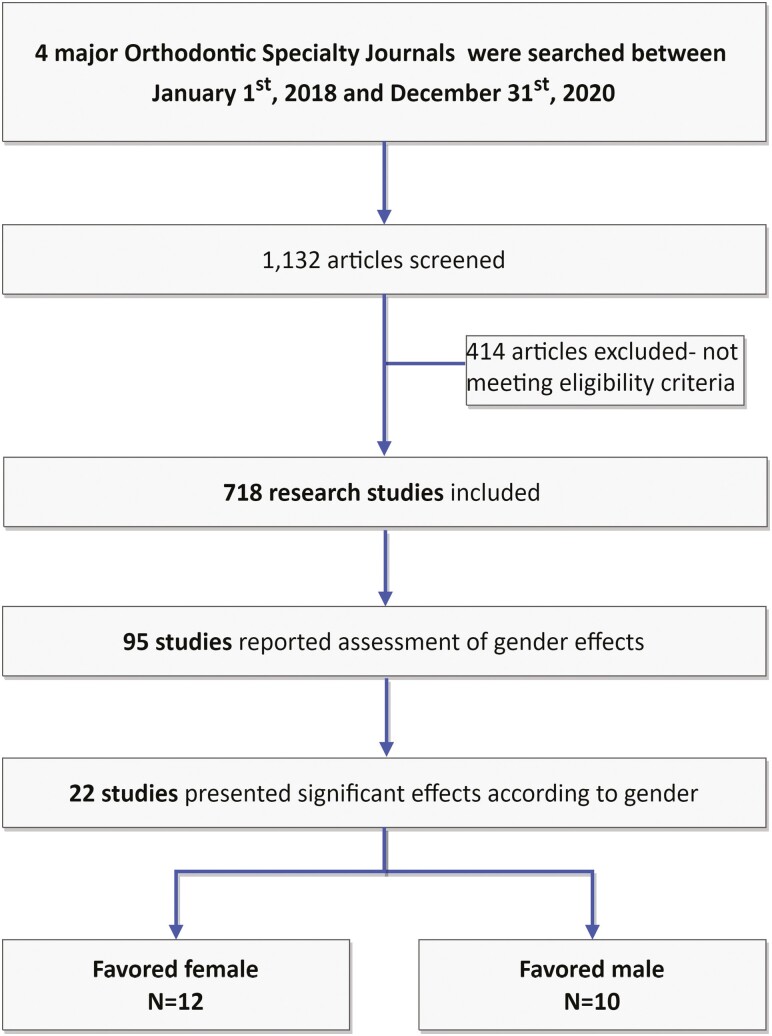
Flowchart of study selection.

A total number of 1,132 articles were screened, after *a priori* exclusion of editorials, letters to the editor, commentaries, and opinions. Of those, a total of 718 papers were eligible for inclusion according to the inclusion criteria. Ninety-five of those papers (13.2%) reported an assessment of impact of gender on treatment effects and relevant outcomes, while 22 out of 95 (23.2%) identified a significant gender effect, either favoring females (*n* = 12), or males (*n* = 10) (Fig. 1).

Descriptively, the pool of 718 included articles were evenly distributed across the years of assessment, while the percentage frequency distribution of articles accounting for gender effect assessment favored the *EJO* (28/154; 18.2%) and the *PIOR* (9/49; 18.4%). Most articles led by investigators originating from European countries implemented assessment of gender-specific effects (45/276; 16.3%), while this was also the case for interventional research designs, either prospective clinical trials or randomized clinical trials (32/186; 17.2%), although the most prevalent type of design met in the assessed sample was that of an observational one (532/718; 74.1%) (Table 1). There was no evidence of a significant effect of any of the assessed publication characteristics (year of publication, journal, continent, and study design) on the odds of including an assessment of gender-specific effects in the analysis of the studies (*P* > .05 in all instances) (Table 2).

**Table 1.** Frequency distribution of recorded publication characteristics for the assessment of gender- specific effects or otherwise (*n* = 718).

	Assessment of gender-specific effects		
	No *N* (%)	Yes *N* (%)	Total *N* (100.0%)
Year of publication			
2018	198 (86.8)	30 (13.2)	228
2019	209 (85.3)	36 (14.7)	245
2020	216 (88.2)	29 (11.8)	245
Journal			
* *AJODO	263 (87.4)	38 (12.6)	301
* *ANGLE	194 (90.7)	20 (9.3)	214
* *EJO	126 (81.8)	28 (18.2)	154
* *PIOR	40 (81.6)	9 (18.4)	49
Continent			
Europe	231 (83.7)	45 (16.3)	276
America	190 (90.9)	19 (9.1)	209
Asia/other	202 (86.7)	31 (13.3)	233
Type of Study Design			
Observational	469 (88.2)	63 (11.8)	532
Interventional	154 (82.8)	32 (17.2)	186
Total	623 (86.8)	95 (13.2)	718

**Table 2.** Univariable and multivariable logistic regression with OR and associated 95% CIs for the effect of a range of predictors on odds of assessing gender-specific effects (*n* = 718).

Category	Univariable			Multivariable		
	OR	95% CI	*P*-value	OR	95% CI	*P*-value
Year of publication			.65^*^			
2018	Reference					
2019	1.14	0.67, 1.92				
2020	0.89	0.51, 1.53				
Journal			.06^*^			.09^*^
* *AJODO	Reference			Reference		
* *ANGLE	0.71	0.40, 1.26		0.70	0.40, 1.25	
* *EJO	1.54	0.90, 2.62		1.47	0.86, 2.52	
* *PIOR	1.56	0.70, 3.46		1.48	0.66, 3.30	
Continent			.07^*^			
Europe	Reference					
America	0.51	0.29, 0.91				
Asia/other	0.79	0.48, 1.29				
Type of Study Design			.06			.10
* *Observational	Reference			Reference		
* *Interventional	1.54	0.97, 2.46		1.48	0.93, 2.36	

^*^Wald test for overall association.

Regarding the sample of 95 identified articles which reported on gender-specific effects’ assessment, the percentage frequency distribution of a significant gender effect for either observational (15/63; 23.8%) or interventional designs (7/32; 21.9%), as well as for outcome type, for adverse being 22.9% (11/48) and for efficacy being 23.4% (11/47), was similar. Studies based on children/adolescent population presented the higher percentage frequency of a significant gender effect (12/41; 29.3%), while a clear majority of articles reporting significant gender effects followed a more sophisticated analysis method, considering gender as a main effect build-in a structured regression model (17/42; 40.5%). Subgroup analyses accounted only for 9.4% of studies (5/53) reporting significant gender effects (Table 3). Breakdown of articles according to intervention categories, revealed that treatment strategies aligned to conventional fixed appliances (9/33, 27.3%), were mostly identified as bearing significant gender effects. The rest were sparsely distributed across other treatment modalities. Regarding comparator groups, the percentage frequency distribution of a significant gender effect was similar for active comparators (11/46; 23.9%) or no comparator at all (9/42; 21.4%). Most studies aimed to assess outcomes such as: tooth movement and duration (20/95; 21.1%), cephalometric-/growth-/anatomical-related outcomes (17/95; 17.9%), material failure (13/95; 13.7%), periodontal-related (11/95; 11.6%) and root resorption (10/95; 10.6%). Of those, root resorption (4/10; 40.0%), tooth movement (6/20; 30.0%), periodontal (3/11; 27.3%) and cephalometric outcomes (4/17; 23.5%) were recorded as those more frequently describing significant gender effects of either side (favoring either female or male). The median sample size for male subjects included in the studies was 27 (interquartile range, IQR: 14–54) and the respective number for female patients was 37 (IQR: 24–74). The difference in sample sizes recruited between genders was statistically significant (signed-rank test: *P* < .001). When frequency distribution for even or uneven sample sizes between genders was assessed, no difference was detected across those presenting significant gender effects or not. As such, significant gender effects were confirmed for 31.6% (6/19) of studies with even gender distribution, while the respective percentage for studies with uneven gender sample distribution was 21.1% (16/76) (χ^2^: *P* = .33) (Table 3). According to the regression analyses, the implementation of structured regression model analyses presented as main effects, with assessment of gender–treatment interaction within the analysis, was associated with 6.53 times higher odds for identifying significant gender effects if those existed (OR = 6.53; 95% CI: 2.15, 19.8; *P* = .001) (Table 4).

**Table 3.** Frequency distribution of recorded variables for the identification of significant gender-specific effects or otherwise (*n* = 95).

	Significant gender-specific effects		
	No *N* (%)	Yes *N* (%)	Total *N* (100.0%)
Type of study design			
Observational	48 (76.2)	15 (23.8)	63
Interventional	25 (78.1)	7 (21.9)	32
Analysis type			
Subgroup	48 (90.6)	5 (9.4)	53
Main	25 (59.5)	17 (40.5)	42
Population			
Adults	24 (82.8)	5 (17.2)	29
Children/adolescents	29 (70.7)	12 (29.3)	41
Mixed	20 (80.0)	5 (20.0)	25
Outcome type			
Adverse	37 (77.1)	11 (22.9)	48
Efficacy	36 (76.6)	11 (23.4)	47
Sample distribution difference according to gender^a^			
No	13 (68.4)	6 (31.6)	19
Yes	60 (79.0)	16 (21.0)	76
Treatment			
Aligners	1 (33.3)	2 (66.7)	3
Bonding method	0 (0.0)	1 (100.0)	1
Essix retainers	6 (100.0)	0 (0.0)	6
Facemask	1 (100.0)	0 (0.0)	1
Fixed appliances	24 (72.7)	9 (27.3)	33
Functional appliances	8 (88.9)	1 (11.1)	9
Headgear	3 (60.0)	2 (40.0)	5
Lingual	1 (100.0)	0 (0.0)	1
Material	5 (100.0)	0 (0.0)	5
Multi-modalities	2 (100.0)	0 (0.0)	2
Non-surgical methods for assistance	1 (50.0)	1 (50.0)	2
Orthognathic surgery	5 (100.0)	0 (0.0)	5
Multi-phase treatment	1 (33.3)	2 (66.7)	3
Segmented fixed approach	0 (0.0)	1 (100.0)	1
Skeletal methods for anchorage	10 (83.3)	2 (16.7)	12
Surgically assisted methods	5 (83.3)	1 (16.7)	6
Comparator			
Active	35 (76.1)	11 (23.9)	46
Untreated control	5 (71.4)	2 (28.6)	7
None	33 (78.6)	9 (21.4)	42
Outcome (actual)			
Caries related	1 (50.0)	1 (50.0)	2
Cephalometric/anatomical/growth measurements	13 (76.5)	4 (23.5)	17
Compliance	3 (100.0)	0 (0.0)	3
Mastication function	4 (100.0)	0 (0.0)	4
Material failure	13 (100.0)	0 (0.0)	13
Occlusion score	1 (100.0)	0 (0.0)	1
Pain	3 (75.0)	1 (25.0)	4
Periodontal related	8 (72.7)	3 (27.3)	11
Psychology/quality of life/satisfaction	6 (66.7)	3 (33.3)	9
Relapse	1 (100.0)	0 (0.0)	1
Root resorption	6 (60.0)	4 (40.0)	10
Tooth movement/duration	14 (70.0)	6 (30.0)	20
Total	73 (76.8)	22 (23.2)	95

^a^A 10% difference in gender enrollment (sample size) was allowed to account for an approximately equal distribution. Exceeding this threshold was indicative of uneven gender distribution.

**Table 4.** Univariable and multivariable logistic regression with OR and associated 95% CIs for the effect of a range of predictors on odds of significant gender-specific effects (*n* = 95).

Category	Univariable			Multivariable		
	OR	95% CI	*P*-value	OR	95% CI	*P*-value
Type of study design			.83			
* *Observational	Reference			Reference		
* *Interventional	0.90	0.32, 2.48				
Analysis type			.001			.001
Subgroup	Reference			Reference		
Main	6.53	2.15, 19.8		6.53	2.15, 19.8	
Population			.46^*^			
Adults	Reference			Reference		
Children/adolescents	1.99	0.61, 6.43				
Mixed	1.20	0.30, 4.74				
Outcome type			.96			
Adverse	Reference			Reference		
Efficacy	1.03	0.40, 2.67				

^*^Wald test for overall association.

**Figure 2.**
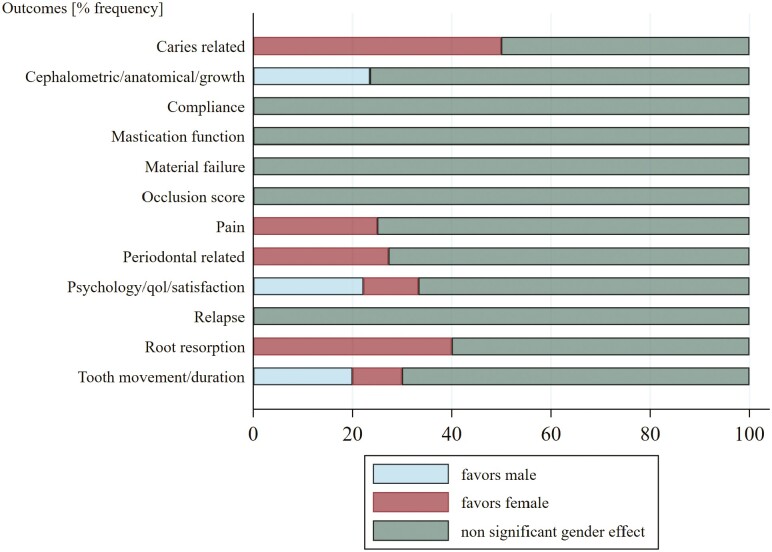
Frequency percentage distribution of gender effect and direction.

A more detailed inspection of the specifics of significant gender conditions, stratified by gender and classified by outcome, is presented in Supplementary Tables 1 and 2). Of note is that all significant gender treatment effects identified for root resorption favored female patients; in essence the respective studies identified more pronounced complications with root resorption in male patients undergoing orthodontic treatment. A similar situation was detected also for periodontal outcomes, where female subjects scored better in terms of bacterial load during orthodontic treatment or development of gingival recession (Supplementary Table 1). Likewise, significant gender outcomes related to treatment effects and reporting on cephalometric and growth-related parameters revealed that male subjects presented a more pronounced gain in this respect (Supplementary Table 2). When considering the wider picture of all 95 studies that reported assessment of gender effects, it was clear that the majority of studies classified by outcome, did not find significant gender-related differences (range of frequency distribution for significant gender differences across all outcomes: 0–40%); an exception was caries in female (50% significant difference), albeit the particularly low number of studies assessing this outcome (Table 3; Fig. 2).

## Discussion

Our findings confirmed that most research articles being published in orthodontics did not pursue for assessment of gender-specific effects of interventions on related outcomes, as a standard research and analysis practice. When this was performed, almost a quarter of studies demonstrated a differential treatment effect on female and male patients. This was highlighted for the first time through the present empirical report, on delivered treatment and intervention strategies which had been utilized in clinical practice for some years; it further shaped an attempt to map respective available evidence on the most prevalent conditions and schemes of therapeutic interventions that have already left a footprint on clinical orthodontics for some time.

Our study findings were aligned with current biomedical evidence and notion that biological and socio-environmental differences attributed to gender have most frequently been neglected upon contemporary medical practice [15]; this evidently impacts diagnosis, prevention and treatment strategies followed upon clinical applications. Efforts have been placed since 2016, when the first Sex and Gender Medical Educations Summit was convened at Mayo Clinic, in Rochester, Minnesota, and also updated in 2021, to incorporate gender issues into medical curricula, increase awareness and integrate knowledge about differences in disease and health, by gender (http://sgbmeducationsummit.com/) [16].

To illustrate the impact of gender-specific health research in medicine, recent studies in the field of cardiology have demonstrated an opposing effect of aspirin in primary treatment of cardiovascular diseases across men and women [17]. In the same line, research in the field of emergency medicine has long been recognized as one that needs improvement of gender-specific medicine adoption [18]. The paradigm of digoxin for the treatment of heart failure overall had initially missed the increased risk of mortality documented only for women [19]. A very recent meta-analysis on anesthesiology concluded on suggestion for potential re-appraisal of anesthetic care guidelines for doses, based on gender [20]. Early evidence based on publications in the *New England Journal of Medicine* across the years 1994 and 1999 revealed that only 14% of trials conducted gender-specific analyses, while the picture was not improved conditional on receipt of funding by the National Institutes of Health [21]. More recent evidence in this respect did not practically reveal any improvement shift for publication practices, with most examined studies not reporting on outcomes by gender or including gender as a covariate for examination in the analysis [22–24].

Interestingly, no similar attempt has been reported in the field of oral health, including orthodontics. A 2022 report on periodontitis-related trials, solely confirmed a frequent disregarding of gender in clinical trial reporting. Additional findings have questioned the level of equity in participant enrollment in clinical trials; in essence, the investigators identified that 25% of examined trials failed to include an approximate equal level of male and female participants, while no further exploration of gender-specific treatment effects and analyses were aimed [25]. A recent orthodontic literature review focused mostly on skeletal, dental, and growth characteristics that differ between the genders. The authors reported that female patients seem to seek orthodontic treatment more often than male although the objective need has been equally distributed in both genders [26]. Moreover, the level of satisfaction pertaining to aesthetic outcomes following orthognathic surgery has been reported to differ with gender [27]. A recent systematic review about early orthopedic treatment of Class III malocclusion revealed evidence of variation in skeletal and dentoalveolar changes between gender, rendering existing evidence unclear to support a definite gender effect [28].

We identified evidence related to gender-specific effects following a thorough investigation of the published orthodontic literature and in essence, we recorded occasions where there were indications of an outcome impacted from gender differences. A clear example was root resorption; male patients undergoing orthodontic treatment with aligners, canine traction mechanics and 1- and 2-phase fixed appliance treatment, were recorded by the respective original studies as more susceptible to root resorption. As a further example in the same line, we also identified certain studies that revealed an increased susceptibility of male patients to periodontal-related issues, such as gingival recessions, following orthodontic treatment. However, it is noteworthy that when considering the wider picture of all 95 studies that included assessment of gender effects in their reporting, it appeared that the majority of studies classified by outcome, did not find significant gender related differences; an exception was caries which favored female patients, nevertheless, this finding could be attributed to chance alone given the respectively small number of studies identified for this outcome. As such, any interpretation or speculation on the identified significant effects should be made with caution, and further documentation of evidence should be provided for the potentially significant gender effects of these outcomes in the future aligned to appropriately designed, conducted, and analyzed studies, to confirm the significance and interpretation of such effects. In any case, the formulated patterns rather suggest a clear basis upon which to build for an upcoming investigation.

Furthermore, if one considers the substantially higher odds for significant gender effects detected by the included original studies when gender was considered as a covariate within the main regression analysis model and/or as a main effect or study objective, it might be that we only spotted a portion of significant gender effects. In this respect, it should also be noted that none of the examined studies was specifically designed to detect gender effects/dimorphism according to primary research question hypothesis reported, however, 13 of 95 studies (13.7%) included inspection of gender effects as a secondary objective. A considerable amount may thus remain undetected and this might constitute a limitation of the present work. Nevertheless, we could solely rely on reporting methodologies of existing studies, rather than actual conduct or re- analysis evidence. The decision to undertake subgroup analyses has been characterized as one potentially bearing well-established limitations, giving rise both to false positive findings, due to multiple testing when multiple covariates are tested, and to false negative due to inadequate statistical power [29, 30]. In the light of the latter, researchers and investigators should move their decisions to undertake such analyses on the basis of hypothesis exploration rather than testing. Clear determination of research objectives, a priori identification of variables subsequently used in subgroup testing, and further reproducibility testing of findings might constitute a fair approach. Thus, identification of true gender effects in treatment outcomes might represent a rather difficult scenario in orthodontic research and beyond. In any case, our empirical study may be regarded as a first step toward the documentation of orthodontic conditions, treatments and outcomes that are long regarded as factors imposing a potentially variable and significant effect of gender; it may be regarded the starting point for future research and quantification of more specific treatment–outcome interactions according to gender. In this respect, the development and universal adoption of a core outcome set, both clinician and patient- centered [31], shall comprise a clear gain toward a holistic interpretation of treatment effects and a more meaningful and effective decision making and treatment planning in the future.

## Conclusions

Only a small fraction of orthodontic research articles included assessment of treatment effects and outcomes according to gender in their design. A quarter of those reported a significantly different effect on the examined outcome, by gender. The majority of outcomes presented non-significant gender effects overall for at least half of the examined studies. On an exploratory basis, patterns of therapeutic effects favoring female and male patients were identified.

## Supplementary Material

cjad073_suppl_Supplementary_Tables_1-2

## Data Availability

The data underlying this article will be shared on reasonable request to the corresponding author.
