# Supplementary material for: Gender-specific treatment effects and outcomes reported in orthodontic research. A cross-sectional empirical study
Source: Eur J Orthod. 2023 Dec 10;46(1):cjad073. doi: 10.1093/ejo/cjad073 (PMC10783147; doi:10.1093/ejo/cjad073)
Supplement: cjad073_suppl_Supplementary_Tables_1-2 [file cjad073_suppl_supplementary_tables_1-2.docx]

**Supplementary Table 1**. List of conditions and outcomes pertaining to significant gender- specific effects, favoring female patients (ie. increased efficacy/ decreased adverse outcomes).

| **Study id** | **condition** | **comparator** | **outcome** |
| --- | --- | --- | --- |
| 729 | orthodontic treatment with clear aligners | none | root resorption |
| 759 | psychosocial impact of dental esthetics | none | psychology/qol/satisfaction |
| 774 | long-term development of gingival recession | untreated control | periodontal |
| 871 | spontaneous angular changes of third molars after mandibular second molar protraction | none | tooth movement/duration |
| 1203 | root resorption of maxillary incisors after orthodontic traction of impacted canines (bilateral vs. unilateral) | active | root resorption |
| 1204 | root resorption of maxillary incisors after orthodontic traction of impacted canines (different levels of complexity) | active | root resorption |
| 1877 | extraction space closing time between two retraction methods | active | tooth movement/duration |
| 2039 | development of white spot lesions in direct or indirect bonding | active | caries |
| 2139 | gingival recession after orthodontic treatment | none | periodontal |
| 2219 | correlation of obesity on orofacial pain during early orthodontic treatment | none | pain |
| 2224 | root resorption in class II divison 2 patients treated with 1- or 2-phase treatment (removable + fixed vs. only fixed) | active | root resorption |
| 3007 | bacterial level during treatment with aligners or fixed | active | periodontal |

qol, quality of life

**Supplementary Table 2**. List of conditions and outcomes pertaining to significant gender- specific effects, favoring male patients (ie. increased efficacy/ decreased adverse outcomes).

| **id** | **condition** | **comparator** | **outcome** |
| --- | --- | --- | --- |
| 763 | effects of vibrational force on space closure with fixed appliances with or without vibrational force supplementation | active | tooth movement/duration |
| 825 | evaluation of treatment time and efficiency for extrusion of impacted maxillary canines | none | tooth movement/duration |
| 886 | 1-phase vs 2-phase trx in Class II patients | active | cephalometric/ anatomical/ growth |
| 1191 | treatment time for active traction of impacted canines | none | tooth movement/duration |
| 2040 | self-esteem before, during and after orthodontic treatment | none | psychology/qol/satisfaction |
| 2045 | oral-health quality of life during orthodontic treatment | none | psychology/qol/satisfaction |
| 2049 | functional appliance treatment in Class II patients (twin block or dynamax) | active | cephalometric/ anatomical/ growth |
| 2079 | facial growth in cleft patients after surgery and control group | untreated control | cephalometric/ anatomical/ growth |
| 2116 | early and later timed treatment with cervical headgear | active | tooth movement/duration |
| 2193 | early and later timed treatment with cervical headgear | active | cephalometric/ anatomical/ growth |

qol, quality of life
